# 

**Published:** 1920-07-03

**Authors:** 


### The College of Nursing
### (Limited by Guarantee).

BRIGHTON AND HOVE CENTRE.

A Picnic to Arundel is being arranged for Saturday, July 17, 1920, but will be held only if a sufficient number of members procure tickets. The char-à-banc will start from the Aquarium at 2.15, and arrive back at the Aquarium about 8.30 p.m. Permission has been given by Her Grace the Duchess of Norfolk for the grounds and castle to be open to members. Tickets will be 6s. 6d. each person, but will not include refreshments. Members may be allowed to take one friend.

Names must be sent in to Miss A. M. Latham, Hon. Secretary, Royal Sussex County Hospital, with a postal order for 6s. 6d., not later than Monday, July 12.

